# Maximal strength training improves muscle-tendon properties and increases tendon matrix remodulation in well-trained triathletes

**DOI:** 10.1038/s41598-025-12721-0

**Published:** 2025-07-27

**Authors:** Mats W. Jacobs, Joshua F. Feuerbacher, Falk Mersmann, Wilhelm Bloch, Adamantios Arampatzis, Moritz Schumann

**Affiliations:** 1 Department of Sports Medicine and Exercise Therapy, Chemnitz University of Technology, Chemnitz, Germany; 2 Department of Molecular and Cellular Sports Medicine, German Sport University Cologne, Cologne, Germany; 3 Department of Training and Movement Sciences, Humboldt-Universität zu Berlin, Berlin, Germany

**Keywords:** Concurrent endurance and strength training, Endurance athletes, Endurance performance, Tendon stiffness, Ultrasound imaging, Extracellular matrix, Physiology, Tendons

## Abstract

This study assessed the effects of a 12-week strength training (ST) intervention on Achilles and patellar tendon stiffness and tendon-matrix blood-borne markers in well-trained triathletes performing concomitant high-volume aerobic training. Eighteen triathletes (VO_2_max: 64.43 ± 4.65 ml kg^−1^ min^−1^) were randomized into a combined endurance and ST group (3-times/week; intervention) or endurance-only group (control). Pre- and post-intervention assessments included ultrasound-based force-elongation measurements of the Achilles and patellar tendons, blood analysis of selected markers before and after an experimental ST session in weeks 1 and 12, and dynamic strength and endurance testing. Achilles (39.1 ± 31.8%) and patellar tendon stiffness (15.8 ± 8.5%), isometric maximal knee extensor strength (8.9 ± 5.7%), and squat one-repetition maximum (20.0 ± 9.7%) significantly increased in the intervention group (all *p* ≤ 0.002). Additionally, serum concentrations of matrix metalloproteinase (MMP) I (46.30 ± 20%; *p* = 0.027) and decorin (30.50 ± 19.94%; *p* = 0.015) increased after the first experimental ST session, while after the second session MMP-III (40.77 ± 20.13%; *p* = 0.040) and decorin (36.59 ± 37.81%; *p* = 0.007) were increased. Baseline tenascin-c concentrations decreased significantly only in the control group (-19.49 ± 20.55%; *p* = 0.029). Our data clearly show improved muscle-tendon properties of the plantar flexor muscles and quadriceps femoris, indicating Achilles and patellar tendon stiffness to be similarly responsive to heavy ST despite concomitantly performing high volumes of endurance training.

**Supplementary Information:**

The online version contains supplementary material available at 10.1038/s41598-025-12721-0.

## Introduction

Previous studies have clearly highlighted the beneficial effects of heavy strength training (ST) for endurance performance^1–5^. While an abundance of mechanisms underlying these effects was previously discussed^1^, particularly the muscle-tendon unit has been of research interest^6,7^. A ST-induced increase in muscle-strength and tendon stiffness can affect the operating strain of tendons during movement and the contractile behaviour of the respective muscles. While from a theoretical perspective an increase in tendon stiffness may reduce tendon strain and, thus, injury risk at a given level of physical activity (see^8,9^ for a mechanistic discussion), the beneficial effects on endurance performance are more clearly established. In this context, cross-sectional associations between Achilles tendon stiffness as well as greater muscle strength of the plantar flexor muscles and running economy (RE)^10^. Moreover, interventional studies have demonstrated that ST-induced increases in Achilles tendon stiffness and plantar flexor muscle strength may be associated with improvements in RE, likely attributed to an enhanced enthalpy efficiency of the soleus muscle during the contact phase of running^6,10^. Interestingly, most of the evidence on how tendon mechanical properties relate to endurance performance originates from studies on the Achilles tendons of endurance runners^6^. Similar to the propulsive role of the plantar flexors during running^11^, the knee extensors contribute most of the mechanical work during cycling and, thus, the patellar tendon has a major influence on their fascicle behavior influencing the shortening velocity^12^. In line with findings in running^6^, it may be hypothesized, that a higher patellar tendon stiffness might improve the contraction behavior of the m. quadriceps femoris, which may result in improved cycling performance (e.g. cycling economy (CE)). However, it remains to be investigated whether the patellar tendon is as responsive as the Achilles tendon to ST in well-trained endurance athletes.

The main stimulus driving the metabolic response and adaptation of tendons is the strain of the tissue and embedded tenocytes under load^13,14^. In vivo data suggests that cyclic high strain magnitudes and long strain durations may be particularly effective for initiating changes in tendon mechanical properties^15,16^. Hereby the mechanical modulation of tendon tissue appears to mainly evolve around an increase in type I collagen synthesis^17^, primarily taking place in the extracellular matrix (ECM). More precisely, especially proteoglycans and glycoproteins are involved in the remodeling process of ECM structure (e.g. collagen I)^18^. For example, the matrix metalloprotease I and III (MMP-I, MMP-III), which initiate the degradation process of type I and III collagens, were shown to be expressed following high-impact exercises^18^. The release of MMP-I and III, in turn, is thought to activate proteoglycans like decorin or thrombospondin-4^19^. However, the role of these proteoglycans in the remodeling process remains not fully understood but it is assumed that their location within the tendon properties will activate growth factors related to the development of collagen I^18^. In this context, decorin appears to work as a mediator of fibril growth and correlates with the size and density of collagen fibers^20^. As a result, glycoproteins like tenascin-c are released and are thought to play a vital role in the actual remodeling process of collagen I structures^21^. The evidence of blood-borne ECM markers, however, remains scarce and whether changes in these blood markers occur as a result of ST in endurance athletes is yet to be investigated.

The primary aim of this study was to assess whether the muscle-tendon properties plantar flexor muscles and m. quadriceps femoris are responsive to heavy ST in well-trained endurance athletes concomitantly performing high volumes of both endurance running and cycling. Furthermore, we aimed to assess if structural changes of these muscle-tendon units are reflected in specific blood-borne markers (i.e. MMP-I & III, decorin, thrombospondin- 4 and tenascin-c) related to the remodeling process of tendon structure. As a secondary aim, we also assessed whether improvements in tendon stiffness and muscle strength would translate into improvements in RE and CE, respectively. We hypothesized that heavy ST increases tendon stiffness and maximal strength of the plantar flexors and m. quadriceps femoris to a similar extent. Furthermore, we hypothesized that the mechanical load of a single ST-session will be reflected in the upregulation of MMP-I & III and proteoglycans (i.e. decorin, thrombospondin-4) as well as a chronic increase of tenascin-c in venous blood.

## Results

Table 1 shows the pre- and post-values of all primary and secondary outcomes. No baseline between-group differences were observed.

**Table 1:** Pre- and post-values for the main outcome variables; post hoc comparison (time effect): * *p* < 0.05. Pmax = Peak power, RE = running economy, CE = cycling economy, VO_2_max = Maximal oxygen consumption, 1 RM = one repetition maximum.

	Intervention group		Control group	
	Pre	Post	Pre	Post
Achilles tendon stiffness [N mm^− 1^]	299.14 ± 46.13	409.60 ± 76.99*	305.85 ± 67.38	287.21 ± 68.81
Patellar tendon stiffness [N mm^− 1^]	1696.17 ± 359.01	1964.06 ± 420.65*	1880.66 ± 333.81	1772.83 ± 359.57
Maximum isometric plantar flexion moment [Nm]	206.49 ± 40.07	224.72 ± 33.72	218.75 ± 33.90	221.86 ± 39.35
Maximum isometric knee extension moment [Nm]	317.02 ± 35.93	345.07 ± 40.44*	305.87 ± 37.70	301.25 ± 40.15
1 RM Squat [kg]	84.67 ± 14.17	100.44 ± 12.30*	78.89 ± 12.91	77.67 ± 12.95
Pmax [W]	397.85 ± 63.03	454.53 ± 64.14*	355.34 ± 61.61	352.07 ± 74.45
RE 2 mmol [kjoule kg^− 1^ km^− 1^]	4.67 ± 0.33	4.59 ± 0.29	4.57 ± 0.32	4.58 ± 0.30
RE 4 mmol [kjoule kg^− 1^ km^− 1^]	4.63 ± 0.27	4.50 ± 0.29	4.51 ± 0.28	4.63 ± 0.30
CE 2mmol [%]	21.15 ± 0.96	20.59 ± 0.56	21.21 ± 0.70	21.10 ± 0.92
CE 4 mmol [%]	21.44 ± 0.85	20.81 ± 0.61	21.23 ± 0.60	21.44 ± 0.84
VO_2_max running [ml min^− 1^ kg^− 1^]	62.19 ± 4.51	61.88 ± 3.88	63.26 ± 2.59	65.49 ± 3.75
VO_2_max cycling [ml min^− 1^ kg^− 1^]	63.35 ± 4.50	63.61 ± 4.53	65.50 ± 4.50	66.62 ± 4.89

### Training characteristics

The endurance training (ET) characteristics are displayed in Table 2. No statistically significant time (*p* = 0.615; *p* = 0.465) or interaction effect (*p* = 0.890; *p* = 0.935) was found for the training impulse method of Morton (TRIMP) or training volume, respectively. Out of 34 planned ST sessions, the intervention group completed a mean of 30.2 ± 3.6 sessions. Except for one participant who missed the first 4 weeks of consecutive training due to illness (23 sessions), all participants completed at least 28 sessions (adherence: 82.3%). In this particular case, an extension of the intervention was not possible due to the beginning of the competition period. As the major changes in tendon stiffness occur during the first 8 weeks of increased mechanical loading^22^, all participants were included in the analysis.

**Table 2:** Endurance training characteristics (training volume & Morton TRIMP) for the intervention and control group. TRIMP = training impulse method.

	Training Volume [h: min]		TRIMP	
	Intervention	Control	Intervention	Control
Week 1–4	07:18 ± 02:01	07:30 ± 02:41	307.69 ± 117.17	357.69 ± 143.91
Week 5–8	08:17 ± 02:11	07:56 ± 02:36	330.13 ± 114.16	338.85 ± 92.85
Week 9–12	08:46 ± 03:17	08:05 ± 03:40	359.20 ± 131.87	361.19 ± 117.79

Although > 8 h/week of training volume was defined as an inclusion criterion to ensure a well-trained and homogeneous sample, individual deviations occurred due to illness-related absences and the fixed duration of the intervention. However, these deviations are considered negligible, as there were no significant differences in training volume or TRIMP between the intervention and control group.

### Muscle strength and tendon mechanical properties

**Fig. 1:**
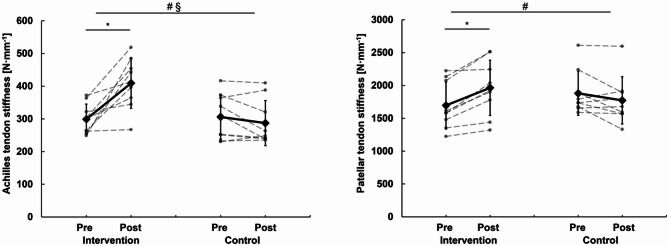
Achilles and patellar tendon stiffness; § significant time effect, # significant interaction effect, *significant post hoc effect.

For Achilles tendon stiffness, significant time (*p* = 0.002) and interaction (*p* < 0.001) effects were found. Achilles tendon stiffness increased significantly in the intervention (39.1 ± 31.8%, *p* = 0.001) but not in the control group (-5.5 ± 12.4%, *p* = 0.270) (Fig. 1). For patellar tendon stiffness, no time (*p* = 0.054) but a significant interaction effect (*p* < 0.001) was observed. Patellar tendon stiffness significantly increased in the intervention (15.8 ± 8.5%, *p* = 0.001) but not in the control group (-5.6 ± 9.9%, *p* = 0.230) (Fig. 1). For the maximum isometric plantar flexion moment, a statistically significant effect was found for time (*p* = 0.025) but not interaction (*p* = 0.295). The post hoc comparisons were neither statistically significant in the intervention (10.5 ± 15.7%, *p* = 0.063) nor the control group (1.2 ± 5.1%, *p* = 0.25). For the maximum isometric knee extension moment, a significant effect was found for time (*p* = 0.003) and interaction (*p* < 0.001). The maximum isometric knee extension moment significantly increased in the intervention (8.9 ± 5.7%, *p* = 0.002) but not in the control group (-1.6 ± 2.8%, *p* = 0.134).

### Dynamic maximal strength

For the 1 RM, significant effects for time (*p* < 0.001) and interaction (*p* < 0.001) were observed. 1 RM increased significantly in the intervention (20.0 ± 9.7%, *p* < 0.001) but not the control group (-1.5 ± 4.1%, *p* = 0.351). Similarly, also for Pmax significant effects for time (*p* = 0.002) and interaction (*p* < 0.001) were found. Pmax increased significantly in the intervention (14.8% ± 8.5%, *p* < 0.001) but not in the control group (-1.2% ± 8.5%, *p* = 0.763).

### Blood-borne marker

Pre and post changes of the blood borne markers throughout both experimental ST sessions are shown in Table 3.

**Table 3:** Acute changes in blood-borne markers throughout both experimental strength training sessions; post hoc comparison (time effect): *p < 0.05. The experimental sessions consisted of 5 lower limb exercises, representing the ST of the intervention period, and were performed in week 1 and week 12 of the intervention period. MMP-I/III = matrix metalloprotease I/III.

	Intervention group			
	Experimental ST session 1		Experimental ST session 2	
	Pre	Post	Pre	Post
MMP-I [ng ml^− 1^]	8.33 ± 2.56	12.22 ± 3.96*	10.38 ± 4.55	10.28 ± 3.81
MMP-III [ng ml^− 1^]	1.93 ± 0.63	2.05 ± 0.59	1.78 ± 0.58	2.43 ± 0.68*
Decorin [pg ml^− 1^]	42.16 ± 8.82	54.17 ± 9.93*	38.78 ± 7.85	50.84 ± 7.59*
Thrombospondin- 4 [ng ml^− 1^]	40.62 ± 8.53	37.49 ± 4.97	38.88 ± 5.04	37.23 ± 7.37

**Fig. 2:**
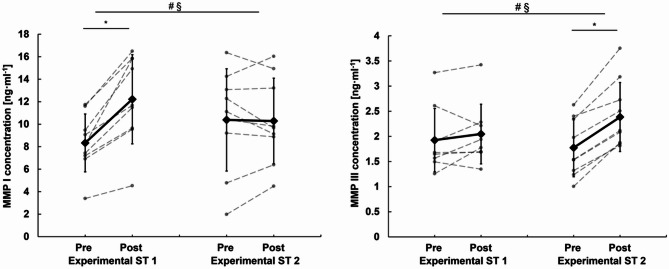
MMP-I and MMP-III concentrations of experimental ST session 1 and 2. The experimental ST sessions were characterized by 5 lower limb exercises and were performed in week 1 and week 12 of the intervention period to asses tendon ECM specific blood borne marker; § significant time effect, # significant interaction effect, *significant post hoc effect. MMP-I/III = matrix metalloprotease I/III.

For the serum concentration of MMP-I, significant effects for time (*p* = 0.003), and interaction (*p* = 0.002) but not group (*p* = 0.956) were observed. MMP-I increased significantly (46.30% ± 20.00%, *p* < 0.001) from pre to post in the first experimental session but remained statistically unchanged during the second experimental ST session (12.97% ± 45.62%, *p* = 0.871) (Fig.2), with the magnitude of MMP-I expression being significantly different between the two experimental ST sessions (*p* < 0.001). For the serum concentration of MMP-III, significant effects for time (*p* < 0.001), and interaction (*p* < 0.001) but not group (*p* = 0.208) were evident. The MMP-III serum concentrations remained statistically unchanged from pre to post during the first experimental ST session (8.44% ± 17.54%, *p* = 0.239) but increased significantly from pre to post during the second experimental ST session (40.77% ± 20.13%, *p* < 0.001) (Fig. 2). Again, the magnitude of the MMP-III expression differed significantly between both experimental ST sessions (*p* < 0.001).

**Fig. 3:**
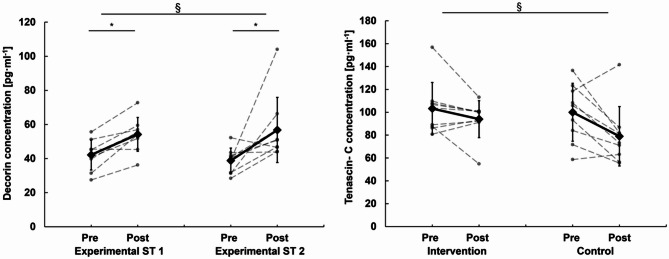
Decorin and tenascin-c concentrations. Decorin concentrations were assessed in experimental ST session 1 and 2. The experimental ST sessions were characterized by 5 lower limb exercises and were performed in week 1 and week 12 of the intervention period to asses tendon ECM specific blood borne marker. Tenascin-c concentration was as the basal concentration of experimental session 1 and 2. § significant time effect, # significant interaction effect, *significant post hoc effect.

Significant effects for the serum concentration of decorin were observed for time (*p* = 0.003) but not group (*p* = 0.568) or interaction (*p* = 0.881). A significant increase from pre to post with similar magnitude was observed following both the experimental ST session 1 (30.50% ± 19.94% (*p* < 0.001) and 2 (36.59% ± 37.81% *p* = 0.026) (Fig. 3). For the serum concentration of thrombospondin-4, significant effects were observed for time (*p* = 0.003) but not group (*p* > 0.05) or interaction (*p* > 0.05). No significant change was found for the thrombospondin-4 concentration in experimental ST session 1 (-6.53% ± 8.24%, *p* = 0.072) and 2 (-4.90% ± 9.32%, *p* = 0.174). A time (*p* = 0.008) but no group (0.355) or interaction effect (*p* = 0.253) was observed for the basal concentrations of tenascin-c serum (Fig. 3). Tenascin-c remained statistically unaltered over the 12-week training period in the intervention group (Pre: 103.21 ± 22.88, Post: 94.01 ± 16.11, Δ: -7.31% ± 16.22%, *p* = 0.163) but significantly decreased in the control group (Pre: 99.98 ± 25.33, Post: 78.99 ± 25.97, Δ: -19.49% ± 20.55%, *p* = 0.029).

### Running and cycling economy

The pre- and post-values for RE and CE of each running velocity and cycling load are shown in supplementary Table 1. For the RE at 2 mmol L^− 1^ and 4 mmol L^− 1^ blood lactate concentration, no significant time (*p* = 0.632 and *p* = 0.834), group (*p* = 0.709 and *p* = 0.961), or interaction effects (*p* = 0.333 and *p* = 0.061) were observed, respectively. Further, no significant time (*p* = 0.123 and *p* = 0.316), group (*p* = 0.387 and *p* = 0.469), or interaction (*p* = 0.294 and *p* = 0.058) effects were found for CE at 2 mmol L^− 1^ and 4 mmol L^− 1^ blood lactate concentration.

## Discussion

The main findings of this study were that 12 weeks of heavy ST led to a significant increase in Achilles and patellar tendon stiffness, isometric plantar flexor and knee extensor strength as well as dynamic strength capacities (i.e. one repetition maximum (1RM) squat, peak power (Pmax) squat) in well-trained triathletes performing high volume endurance training at the same time. Furthermore, significant changes in serum concentrations of MMP-I following the initial and MMP-III following the second experimental ST session as well as decorin following both experimental ST sessions were observed. Chronic changes in tenascin-c were observed in blood serum concentrations, showing a significant decrease in the control group. RE and CE remained statistically unaltered in both the control and intervention groups.

The observed increases in Achilles tendon stiffness in the intervention group are well in line with previous data obtained following sole strength training^6,15,23^. Our data confirm that, in contrast to sole endurance running and cycling^24,25^, heavy ST with long times under tension seems to provide an appropriate stimulus in terms of strain magnitude and duration to trigger an adaptation of tendon mechanical properties^15,16^. The observed magnitude of adaptations are also in line with previous interventional studies, demonstrating increased Achilles tendon stiffness in recreational long-distance runners following specific strength training of the plantar flexors^6,10^. However, the endurance training volume in these previous studies was lower than that of the triathletes included in the present investigation, highlighting that tendon stiffness can also be increased when concomitantly performing high volumes of aerobic training. Moreover, while these previous studies focused only on the Achilles tendon and performing ST in athletes specialized in running, our data indicates ST can increase not only the Achilles but also the patellar tendon stiffness. This finding expands on previous data that suggested that morphological changes (i.e. increased cross sectional area (CSA)) of the patellar tendon following combined endurance and strength training in a group of well-trained cyclists may be blunted^26^. However, it is a common observation in adults that tendon morphological changes in response to mechanical loading are rather small as opposed to their material properties^27^ and in our current understanding the effect of tendons on the function of the muscle-tendon unit is determined by tendon stiffness and not CSA^28,29^. By this, significant associations between tendon stiffness and the rate of torque development as well as the jump height in squat jumps and countermovement jumps were shown previously^30^. In line with that, along with improvements in Achilles and patellar tendon stiffness, we also observed significant improvements in maximal strength (i.e. 1RM and Pmax). While this may at least partly be explained by an improved force velocity potential resulting from increased tendon stiffness^30^, especially in endurance athletes without a history of structured strength training, it cannot be ruled out that these improvements might be rather related to neural adaptations (e.g., improved motor unit recruitment), muscle architectural changes (e.g., increased fascicle length), and hypertrophy, all of which contribute to improved mechanical efficiency and force transmission^31,32^. Whether increased tendon stiffness might directly influence cycling performance remains to be investigated further.

The changes in mechanical properties of the Achilles and Patellar tendons were associated with acute ST-induced increases in blood-borne markers related to the tendon ECM. More precisely, we found a significant increase of MMP-I following the first and an elevated MMP-III serum concentration following the second experimental ST session. Previous research has shown increased serum concentrations of MMP-I & III as a result of an acute mechanical stimulus only in untrained men^33^. Our findings indicate that remodeling processes of the tendon ECM appear to occur in endurance trained men performing heavy ST as well. Further, it was demonstrated that already 7–14 days of stimulation resulted in a significantly increased mRNA expression of MMP-I and MMP-III^34^whereby the magnitude of MMP-I was greater than that of MMP-III, which we also observed in our data. However, since changes in serum concentrations of blood-borne markers might be influenced by sources other than tendons a direct comparison between changes in mRNA and blood serum is difficult. Moreover, why MMP-I was increased only after the initial but not the second ST session and vice versa for MMP-III remains unknown and should be explored in future studies. It should also be noted that the analysis of the expression of these blood-borne markers was limited to the acute response to two resistance training sessions and no comparison to a control condition was included.

Additionally, we showed that the proteoglycan decorin was significantly increased following both experimental ST sessions in week 1 and week 12, respectively. Since proteoglycans play an important role in connecting fibrous elements in ECM^18^, an increase in serum concentrations of decorin was somewhat expected. Moreover, decorin in particular is known to envelope the collagen fibrils^18^ which leads to maintaining the tendon integrity at molecular level. Hence, an increased concentration may indicate tendon remodulation of existing collagen structures following acute mechanical stimuli. As a result, it is assumed that decorin could work as a mediator of fibril growth and is released from the tendon tissue to the blood stream as a reaction to an increased MMP concentration^18^. However, since the exact role of proteoglycans like decorin is not fully understood, a more precise interpretation remains speculative. The reason behind the missing change in thrombospondin-4 following the acute ST stimulus remains inconclusive. Thrombospondin-4 is known to regulate the interaction between proteins or proteins and cells within the ECM^35^ but seems not to be directly involved into the structural processes of the tendon ECM. Thus, it might be hypothesized that the acute ST was not sufficient to result in a significant expression. Interestingly, we found a significant chronic decrease in serum concentrations of tenascin-c in the control group, while it remained statistically unchanged in the intervention group. Since tenascin-c seems to mediate the actual synthesis of new collagen fibers and is, therefore, indicative for regenerative processes within the tendon, the 12-week ST intervention theoretically should have led to an increase in the intervention group. Moreover, previous research already showed an increase in tenascin-c concentration following a chronic ST stimulus^34^, thus the lack of increased serum levels in the intervention group of this study is somewhat surprising. However, while chronic mechanical stimuli on the muscle-tendon properties regulate tenascin-c expression, the timing of measurement in this study might have influenced our findings. It needs to be noted that the intervention started right after the participants ended their competition phase, where the chronic load on muscle-tendon properties was high. Following this period, the control group reduced the mechanical stimuli on the muscle-tendon properties by just completing, mostly low-intensity, endurance training, which might explain the significant decrease in tenascin-c. Contrary, due to the additional ST performed 3 times a week in the intervention group, the mechanical stimulus might have been sufficient enough to maintain tenascin-c serum levels, potentially initiating further remodulation processes of the tendon structure and thereby explaining the lack of statistical change. Taken together with the significant acute expression of MMP-I, MMP-III and decorin, these acute and chronic changes at the molecular level are supportive of a remodulation of the Achilles and patellar tendon structure.

Although we were able to demonstrate significant acute responses (i.e. ECM marker expression) and mechanical adaptation of the tendon (i.e. tendon stiffness) as well as an increase in muscle strength following heavy ST, we did not find a transfer into enhanced endurance performance. This is somewhat in contrast to previous studies that have shown significant associations between changes in Achilles tendon stiffness and running economy^3,5,6^. Since no previous study assessed how changes in patellar tendon stiffness relates to CE, an interpretation of the missing changes in this study remains highly speculative. However, it might be hypothesized that because of the lower ratio of tendon to muscle length of the patellar tendon and m. quadriceps femoris, the potential effects of increased tendon stiffness on muscle fibre behaviour and CE might be smaller compared to the Achilles tendon and plantar flexor muscles.

### Limitations

Some limitations have to be considered when interpreting the findings of this study. Since the main objective of this study was to investigate tendon adaptations in multidisciplinary endurance athletes with a high training volume, it cannot be ruled out that the lack of changes in RE and CE may be mostly related to low statistical power. Indeed, the a priori analysis was done for expected adaptations of tendon properties rather than changes in RE or CE. Furthermore, improvements in RE and CE following ST can be influenced by a variety of factors (e.g. tendon stiffness, recruitment pattern, muscle fiber type), which were only partly analyzed in this study. Furthermore, although the intervention period was timed during the general preparation phase where training volume and intensity distribution remained quite stable, it cannot be ruled out completely that individual differences in the training program between the athletes might have influenced the adaptations in RE and CE.

## Conclusion

Our data show that ST may induce structural and functional improvements of the muscle-tendon complex of the plantar flexors and m. quadriceps femoris in well-trained triathletes. The increases in Achilles and patellar tendon stiffness were accompanied by acute ST-induced increases in MMP-I, MMP-III and decorin, while chronic reductions in tenascin-c concentrations were observed in the control group. These results indicate that ST provides a sufficient stimulus to initiate acute molecular expressions of blood-borne markers in endurance athletes, potentially mediating the observed adaptations in tendon mechanical properties. Thus, our data provide novel insights into the beneficial effects of mechanical stimuli on the expression of ECM markers that may be associated with the structural remodeling of tendons in well-trained endurance athletes performing a high endurance training volume. Even though in the present study the tendon remodeling was not associated with movement economy, the increased Achilles and patellar tendon stiffness may well be correlated with other benefits, such as a reduction in risk of injury. However, such associations were beyond the scope of the present study and should be explored in future investigations.

## Materials and methods

### Participants

The sample size was set a priori, using the main outcome of increased Achilles tendon stiffness. The required sample size was calculated using G*Power 3.1^37^ with α = 0.05 and an anticipated moderate effect (f = 0.25) of the intervention based on previous data^6^. The calculation resulted in a projected sample size of 16 participants (8 per group).

To account for possible drop-outs, 18 well-trained male triathletes (age: 26 ± 6 years; height: 184.7 ± 6.3 cm; body mass: 75.12 ± 5.45 kg; running maximal oxygen consumption (VO_2_max): 62.75 ± 3.72 ml kg^− 1^ min^− 1^; cycling VO_2_max: 64.58 ± 4.75 ml kg^− 1^ min^− 1^) were recruited to participate in this study. Inclusion criteria included three or more years of triathlon experience, competing at least at a regional level and a minimum of 8 h of weekly endurance training volume. Exclusion criteria included previous structured heavy ST or a history of Achilles or patellar tendon injury. All participants were informed about the potential risks associated with this study and completed a medical questionnaire prior to inclusion. Furthermore, written informed consent was received from all participants before enrolment. The study was approved by the local ethic committee of the German Sport University Cologne (170/2022) and was performed in accordance with the Declaration of Helsinki.

### Experimental design

**Fig. 4:**
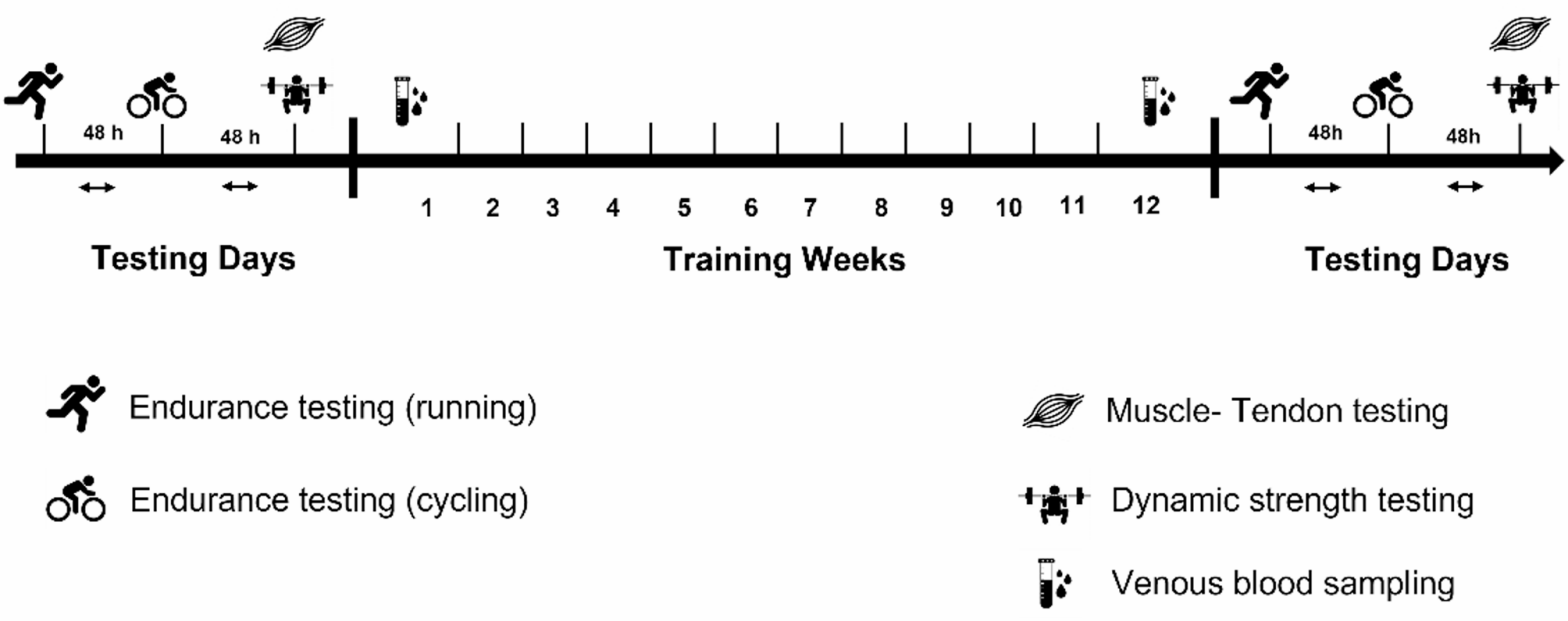
Study design. Muscle-tendon testing consisted of ultrasound imaging of the Achilles and Patellar tendons as well as isometric strength testing of plantar flexors and m.quadriceps femoris. Dynamic strength testing consisted of a graded 1 RM test of the dynamic squat exercise. Endurance testing in running and cycling consisted of both a graded step and ramp test. Venous blood sampling was performed in two experimental sessions to collect specific blood-borne markers of tendon ECM.

The study had a randomized-controlled design, including a 12-week training intervention. Testing consisted of combined ultrasound and dynamometry to determine Achilles and patellar tendon stiffness, isometric strength testing to assess plantar flexor and knee extensor muscle strength, an incremental maximal strength test to evaluate individual strength capacities, venous blood sampling for the analysis of chronic and acute changes of ECM blood-borne markers (i.e. MMP-I & III, decorin, thrombospondin- 4, tenascin-c) as well as a graded running and cycling tests to assess RE and CE (Fig. 4). Following baseline testing, the participants were randomized into an intervention- and control group. Randomization into groups was performed with RITA (Randomization in Treatment Arms, Version 1.51, A. Ziegler & I.R. König) using previous training characteristics (TRIMP), isometric maximal strength of the knee extensors and the VO_2_max as stratification criteria. The control group continued with regular ET, while the intervention group performed additional heavy ST 3 times per week.

### Testing

#### Tendon mechanical properties and isometric strength testing

The mechanical properties of the Achilles and patellar tendons as well as the maximum isometric muscle strength of the plantar flexors and the knee extensors were measured using a custom-built transportable diagnostic system^38^. After familiarization, the participants performed three maximal voluntary isometric contractions of the plantar flexors and knee extensors, respectively. The isometric knee extensions were performed at a knee joint angle of 60° (measured with a goniometer at the plateau of the contraction) against a non-elastic rope that was fixed in series with a force sensor (2 kN; Biovision, Wehrheim, Germany) perpendicular to the shank. The force data were recorded at 200 Hz and low-pass filtered with a second-order Butterworth filter (cut-off frequency of 6 Hz) and multiplied with the distance from the lateral epicondyle (as assumed axis of rotation) to the point of force application at the shank to obtain the knee joint moments. During the isometric plantar flexion contractions, the knee joint was fully extended, and the ankle was positioned in neutral (0°; sole of the foot perpendicular to the shank). Resistance during the contractions was provided by means of a harness around the hip, fixed with the portable device via a rigid lashing strap. The foot was plantarflexed with maximum effort against a Wii Balance Board (Nintendo, Kyoto, Japan), which shows good accuracy for the measurement of force and center of pressure (CoP; errors of ± 9 N and 4 mm, respectively) and excellent within-device reliability (4.5 N and 1.5 mm;^39^). The ankle joint moments were calculated considering the instantaneous moment arm of the applied force, which was defined as the anteposterior distance from the CoP to the medial malleolus (i.e. assumed location of the rotation axis). The relative position of the CoP with regard to the medial malleolus was calculated based on the assumption that the CoP would be located at the level of the first metatarsal-phalangeal joint (MPJ) during the plateau of the contraction as follows:

$$\:{\varvec{d}}_{\varvec{i}}\:=\:\varvec{c}\varvec{o}\varvec{s}\:{\varvec{\alpha\:}}_{\varvec{i}}\:\bullet\:\:{\varvec{l}}_{\varvec{r}\varvec{e}\varvec{s}\varvec{t}}-\:{\varvec{Y}}_{\varvec{m}\varvec{a}\varvec{x}}\:-\:{\varvec{Y}}_{\varvec{i}}$$

with *d*_*i*_ being the moment arm and α_i_ the ankle joint angle at time point *i*, *l*_*rest*_ the distance from the MPJ to the medial malleolus arm measured at rest, Ymax the maximum position (i.e. at the plateau of the contraction) and Yi the instantaneous position of the CoP on the force plate Y-axis (which was aligned with the longitudinal foot axis). This calculation considers the change in this moment arm due to the inevitable plantar flexion (and resultant displacement of the rotation axis) during the isometric contractions. The respective angular rotation (13 ± 5°) was measured using two accelerometers (Biovision) placed over the 2nd metatarsal bone and the tibia. The maximum joint moment of the three respective trials of knee extension and plantar flexion served as a measure of maximum strength.

To determine the mechanical properties of the Achilles and patellar tendon, three additional isometric ramp contractions (i.e. a steady increase in force from rest to maximum in 5 s) were performed with real-time feedback on the instantaneous forces generated and a target curve for the force progression. A 10 cm wide linear ultrasound probe (7.5 MHz, Esaote MyLab 60) was used to capture the elongation of the patellar tendon and the displacement of the gastrocnemius medialis myotendinous junction (GM-MTJ), respectively. The analysis of the ultrasound sequences and measurements of tendon rest length were described earlier in detail (e.g.^49,47^ and the average measurements of three trials were taken to achieve high reliability (ICC > 0.9;^40^). To account for the displacement of the GM-MTJ due to the inevitable ankle angle changes during the ramp contractions, an additional passive rotation of the ankle joint was performed manually by an investigator, and the resultant displacement as a function of ankle angle was subtracted from the displacements measured during the isometric trials^41^. For the calculation of tendon forces, the moment arm of the patellar tendon was approximated based on anthropometry^42^, while the moment arm of the Achilles tendon was calculated as perpendicular distance between the anteroposterior center of the tendon (determined in relation to the skin surface based on a sagittal plane ultrasound scan) and the assumed axis of rotation between the malleoli as described elsewhere^43^. In this study, however, the distances between the two malleoli and the skin surface were measured at the proximodistal level of the malleoli during upright stance using a caliper. For both tendons, the same moment arm was used for the calculations in the pre and post assessment, as, to our knowledge, training-induced changes are likely lower than for example the average error for repeated measures of tendon thickness^44^). The three resultant force elongation curves of both tendons were averaged and fitted using a second order polynomial function. Stiffness was then calculated as quotient of the change in force and elongation between 50 and 80% of the fitted curve.

### Dynamic strength testing

The maximal dynamic strength capacities were assessed using a modified graded 1 RM test which included the assessment of the movement velocities over a range of different loads^45^. After performing three repetitions with the initial load of 20 kg, the load was increased and the number of repetitions decreased depending on the individual maximum velocity (MV) of the participants. The following load scheme was applied depending on the MV:

- MV > 1.0 m s^-1^: +10 kg with 3 repetitions.
- MV 1.0–0.5 m s^-1^: +5 kg with 2 repetitions.
- MV < 0.5 m s^-1^: +2.5 kg with 1 repetition.

For every repetition, MV was recorded using a linear velocity transducer (GymAware RS, Kinetic Performance Technology, Bradon, Australia). The participants were instructed to perform the concentric part of the movement as fast as possible. To minimize potential effects of the stretch-shortening cycle, a pause of approximately 0.5 s was implemented at the turning point of the squat. The onset of the concentric phase was initiated by an acoustic signal. The 1 RM was defined by the maximal load [kg] carried out by the participant without external help. The peak power (Pmax) [W] was obtained from the load velocity profile of the squat.

### Endurance testing

Graded running and cycling tests were performed on 2 separate days with a minimum of 48 h in between. Both, the running and cycling protocols consisted of an incremental step test to assess economy at different velocities and loads, followed by an incremental ramp protocol to assess VO_2_max. The running protocol was performed on a treadmill (pulsar^®^ 3p, h/p/cosmos sports & medical GmbH, Nussdorf-Traunstein, Germany) starting at 2.4 m s^− 1^ with a 1% incline. The running speed was increased by 0.4 m s^− 1^ every 5 min. The treadmill was stopped for 30 s every 5 min for the collection of blood lactate samples. If the lactate concentration of the previous velocity was ≥ 4 mmol L^− 1^, the test was terminated. Participants were then required to perform a 5-minute active cool-down at 2.4 m s^− 1^ and a further 10 min of passive recovery before starting the ramp protocol which again commenced at a running velocity of 2.4 m s^− 1^. Every minute, the running velocity increased by 0.4 m s^− 1^ and the test was terminated at voluntary exhaustion of the participants.

The cycling test was carried out on a cycle ergometer (SRM Ergometer, SRM International SRM GmbH, Jülich, Germany). The test commenced at 150 Watts and increased by 30 watts every 5 min in the step test and every 1 min in the ramp protocol, respectively. Capillary blood samples for the assessment of blood lactate concentrations were collected during the final 30 s of each stage. Similar to the running test, once the lactate concentration reached 4 mmol L^− 1^, the test was terminated and the participants were required to perform a 5-minute active cool down at 150 W followed by 10 min of passive recovery before starting the ramp protocol.

Blood lactate concentrations were determined from 20 µL capillary blood sampled from the earlobe. The samples were analyzed using a Biosen C-line analyzer (EKF-diagnostic GmbH, Barleben, Germany).

Breathing gases were continuously measured throughout the step and ramp protocols (Metalyzer 3B, CORTEX Biophysik GmbH, Leipzig, Germany) to determine RE, CE, and VO_2_max. RE and CE were analyzed for individual running velocities and cycling loads at 2 and 4 mmol L^− 1^ blood lactate concentration. To ensure a steady state of oxygen consumption during submaximal running and cycling, the mean of each minute of the 5-minute intervals was calculated and a cut-off value of 5% difference between the individual 1-minute values was defined. All participants showed a steady state for the last 3 min of each increment (changes < 5%). RE was expressed as energy expenditure (kjoule kg^− 1^ km^− 1^) following the equation of Fletcher and colleagues^46^, while CE was expressed as the quotient of power and metabolic cost (gross efficiency [%]).VO_2_max was calculated as the highest 30-second rolling average of the ramp test and was presented as ml kg^− 1^ min^− 1^.

### Blood sampling and analyses

Blood sampling in the intervention group was performed before (i.e. baseline) and after the third (week 1) and last training session (week 12). This included the analysis of concentrations of MMP-I, MMP-III, decorin and thrombospondin- 4. The respective blood borne markers where chosen, to cover the regulatory process in tendon remodeling (proteoglycans- glycoproteins- type1 collagen synthesis). Since in the control group no ST was performed, only baseline samples were collected in weeks 1 and 12, respectively. This included the assessment of tenascin-c concentrations and was compared between the control and intervention groups. Participants reported to the laboratory in a fasted state for the first blood sample. Following this, participants of the intervention group were allowed to consume a self-selected breakfast (containing mainly carbohydrates) before performing a standardized ST protocol. The composition of food consumed was recorded and maintained during the second measurement in week 12. The post-ST sample for the acute analysis was collected immediately after completing the training session (< 1 min). Experimental ST sessions in weeks 1 and 12 aimed to represent the overall ST program and, therefore, consisted of unilateral leg extension, unilateral plantarflexion, bilateral squat, bilateral leg press, and bilateral leg flexion. The unilateral leg extension and plantar flexion included 3 sets with 5 RM and the bilateral exercises of 3 sets with 8 RM. All exercises comprised a 2-minute interset-rest. To ensure a high mechanical stimulus, each repetition was performed with 1 s of knee extension/plantarflexion and 2 s of knee flexion/dorsiflexion.

The blood was collected from the antecubital vein using sterile Vacutainers (BD Vacutainer, Beckton, Dickinson, Heidelberg, Germany) in serum separation tubes and centrifuged 10 min after collection (3500 RPM). The serum was aliquoted and stored at -80 °C for further analysis. Accordingly, the serum concentrations of MMP-I (ELISA Kit, P08254, Invitrogen ThermoFisher Scientific, Massachusetts, USA), MMP-III (ELISA Kit, P03956, Invitrogen ThermoFisher Scientific, Massachusetts, USA) decorin (ELISA KIT, RAB0140, Sigma-Aldrich, Missouri, USA) and thrombospondin-4 (ELISA Kit, ABIN6959934, antibodies-online GmbH, Germany) were assessed by enzyme-linked immunosorbent assay (ELISA). As a marker of chronic modulations of tendon properties, concentrations of tenascin-c were assessed (ELISA Kit, P24821, Invitrogen ThermoFisher Scientific, Massachusetts, USA). Samples were analyzed in duplicate. The intra-assay CV was as follows: MMP- I—5.46%, MMP- III—8.14%, decorin − 9.07%, thrombospondin- 4 —4.90%, tenascin-c – 4.96%.

### Strength training

The heavy ST consisted of five exercises performed three times per week in addition to the ET, with at least 48 h between subsequent ST sessions. To minimize the fatiguing effects of previous ET, ST was performed before ET or after a rest of at least 8 h. ST targeted the lower limbs and comprised of unilateral plantar flexions and knee extensions as well as bilateral free weighted squats, leg press, and leg curls (the detailed strength training program is shown in supplementary Table2). Briefly, the ST focused on maximal strength and consisted of 3–10 repetitions at ~ 80–90% of the 1 RM (based on the number of repetitions performed). After a 10-minute warm-up, participants began with the unilateral and continued with the bilateral exercises. Since a time under tension of ~ 3 s was shown to be favorable for adaptations of the tendon^47^, the loading targeted for a 1-second shortening and a 2-second lengthening phase of the respective muscles. A 3-minute rest period was implemented between each set. To ensure a high stimulus on muscle and tendon structures, the ST load for each participant was based on repetition maxima. The starting weight of the first set served as the basis for determining subsequent loads of this particular training session, so that a maximal stimulus was ensured for each exercises regardless of day-to-day variances of the participants. The aim was to achieve the maximal load for the intended number of repetitions to ensure the high strain magnitude that is crucial for the adaptation of tendon properties^15,16^. To account for general training adaptations, the individual absolute loads were increased by 2.5% every 2 weeks.

### Endurance training

The participants’ ET routine was self-selected during the intervention period. However, to minimize potential influences on the outcome parameters they were instructed to maintain the overall volume and intensity of their ET (including running, cycling, and swimming) during the intervention period. Hence, the training characteristics of each participant were continuously monitored by means of training volume (minutes) and heart rate for both running and cycling, respectively. Since it is not typical to assess the heart rate during swimming sessions, only training volume was monitored. Training data were collected weekly and included the training intensity distribution based on the time-in-zone approach, and TRIMP^48^. In case of major deviations for TRIMP or training volume in the weekly analysis, participants were encouraged to adjust their training volume, intensity distribution, or both. However, due to the general preparation phase of the athletes, the intensity distribution and training volume remained individually stable.

### Statistics

The statistical analysis was performed using R-Studio (version 2022.02.3 + 492, Posit PBC, Boston, USA). Normality of distribution was assessed by the Shapiro-Wilk test for all variables. A mixed factorial analysis of variance with repeated measures (time: T0, T1; group: Intervention vs. Control) was then used to analyze differences in tendon stiffness, muscle strength, tenascin-c concentration (primary aim) as well as RE and CE (secondary aim) (package: rstatix). In case a main time or interaction effect was observed, additionally, post hoc tests were performed using a pairwise t-test. Acute changes in serum concentrations for the blood-borne markers MMP-I & III, decorin and thrombospondin-4 in the intervention group were compared within an experimental ST session (i.e. time) as well as between the two experimental ST sessions (i.e. group) via a repeated measure ANOVA with two within factors (acute ST session [group], timepoint [time]) (package: rstatix). Subsequently, a post hoc test was performed using a pairwise t-test. If an interaction effect between the magnitude of blood-borne marker expression (time) and the experimental ST session (group) was observed, the magnitude of the blood-borne marker expression in each experimental session was calculated and differences were analyzed using a paired t-test. The level of significance for all tests was set to α = 0.05. Data are presented as mean ± SD.

## Electronic supplementary material

Below is the link to the electronic supplementary material.

Supplementary Material 1

## Data Availability

Data are available by the corresponding author upon reasonable request. This includes, for example, further scientific processing or statistical verification.
