# Supplementary material for: Maximal strength training improves muscle-tendon properties and increases tendon matrix remodulation in well-trained triathletes
Source: Sci Rep. 2025 Jul 27;15:27333. doi: 10.1038/s41598-025-12721-0 (PMC12301477; doi:10.1038/s41598-025-12721-0)
Supplement: Supplementary file 1 — Supplementary Material 1 [file 41598_2025_12721_MOESM1_ESM.docx]

Supplementary table 1: Running- and Cycling economy for each running velocity or cycling load. The running economy is displayed as energy cost [kjoule∙kg^-1^∙km^-1^] and the cycling economy as gross efficiency [%].

|  | Intervention | | Control | |
| --- | --- | --- | --- | --- |
| RE | Pre | Post | Pre | Post |
| 2.4 | 4.88 ± 0.38 | 4.82 ± 0.38 | 4.72 ± 0.29 | 4.71 ± 0.48 |
| 2.8 | 4.74 ± 0.36 | 4.61 ± 0.35 | 4.56 ± 0.25 | 4.66 ± 0.33 |
| 3.2 | 4.67 ± 0.33 | 4.55 ± 0.35 | 4.47 ± 0.22 | 4.55 ± 0.33 |
| 3.6 | 4.65 ± 0.34 | 4.48 ± 0.39 | 4.49 ± 0.21 | 4.55 ± 0.35 |
| 4.0 | 4.58 ± 0.27 | 4.50 ± 0.29 | 4.48 ± 0.22 | 4.60 ± 0.29 |
|  |  |  |  |  |
|  | Intervention | | Control | |
| CE | Pre | Post | Pre | Post |
| 150 | 19.84 ± 0.67 | 19.74 ± 0.86 | 19.89 ± 0.61 | 19.84 ± 0.94 |
| 180 | 20.72 ± 0.79 | 20.44 ± 0.75 | 20.64 ± 0.63 | 20.48 ± 0.83 |
| 210 | 20.99 ± 0.88 | 20.54 ± 0.49 | 20.89 ± 0.86 | 20.57 ± 0.56 |
| 240 | 21.47 ± 0.84 | 20.97 ± 0.61 | 21.60 ± 0.84 | 20.99 ± 0.65 |
| 270 | 21.49 ± 0.80 | 21.10 ± 0.49 | 21.53 ± 0.86 | 20.96 ± 0.39 |

Supplementary table 2: Strength training regime over the 12 weeks training intervention incorporating set and repetition structure for the unilateral exercises (upper part) and the bilateral exercises (lower part).

| **Week** | **Load** | | |
| --- | --- | --- | --- |
|  | Session 1 | Session 2 | Session 3 |
| 1 – 4 | 3 x 8 RM | 3 x 5 RM | 3 x 8 RM |
| 5 – 8 | 3 x 5 RM | 3 x 3 RM | 3 x 5 RM |
| 9 – 12 | 3 x 3 RM | 3 x 3 RM | 3 x 3 RM |

| **Week** | **Load** | | |
| --- | --- | --- | --- |
|  | Session 1 | Session 2 | Session 3 |
| 1 – 4 | 3 x 10 RM | 3 x 8 RM | 3 x 10 RM |
| 5 – 8 | 3 x 8 RM | 3 x 5 RM | 3 x 8 RM |
| 9 – 12 | 3 x 5 RM | 3 x 3 RM | 3 x 5 RM |
